# Stakeholder Perspectives on Ear and Hearing Health Service Provision for Aboriginal and Torres Strait Islander Children

**DOI:** 10.1111/ajr.70167

**Published:** 2026-03-25

**Authors:** Jacqueline H. Stephens, Brianna F. Poirier, Amanda Machell, Patricia L. Macfarlane, Nicola J. Spurrier, Leanne Quirino

**Affiliations:** ^1^ Flinders Health and Medical Research Institute, College of Medicine and Public Health Flinders University Adelaide Australia; ^2^ Indigenous Oral Health Unit University of Adelaide Adelaide Australia; ^3^ Department of Otolaryngology, Head and Neck Surgery Flinders Medical Centre Adelaide Australia; ^4^ Department of Health and Wellbeing Adelaide Australia; ^5^ Aboriginal Health Council of South Australia Adelaide Australia

**Keywords:** Aboriginal and Torres Strait Islander health, Audiology, Community Health Workers, Health Services, Otitis media

## Abstract

**Objective:**

Ear disease and hearing loss are largely preventable; however, Aboriginal and Torres Strait Islander children experience some of the highest rates globally. National and state guidelines recommend involving Aboriginal Health Workers/Practitioners (AHWP) in ear and hearing screening, but no investigation has been conducted into stakeholder perspectives on their role.

**Setting, Participants and Design:**

Key stakeholders involved in ear and hearing screening participated in semi‐structured interviews via teleconference to explore their views on current screening processes, AHWP engagement and strategies for improvement.

**Results:**

Thirteen stakeholders participated. Five themes were identified: (i) a fragmented, short‐sighted and inconsistent system; (ii) confusion around scope of practice; (iii) workforce capacity; (iv) community awareness and knowledge and (v) service provision and utilisation. Strategies for improvement included strengthening workforce capacity, implementing a family friendly approach (e.g., no age limits), dedicated ear health champions to facilitate timely and appropriate follow‐up and the potential role for technology in the ear health pathway. Stakeholders also advocated for the need for community education to raise awareness of the impacts of poor ear and hearing health.

**Conclusions:**

This study describes the current state of ear and hearing health services for Aboriginal children and provides insights into improving ear and hearing screening programmes.

## Boxed Lists

What is already known on this subject:

- Colonisation has intentionally disrupted Aboriginal and Torres Strait Islander culture, health and wellbeing.
- Aboriginal and Torres Strait Islander children in Australia experience some of the highest levels of ear disease globally.
- Current approaches are not addressing the persistent high rates of ear disease amongst Aboriginal and Torres Strait Islander children.

What this paper adds:

- Stakeholders identified dedicated ear health champions to aid in facilitating timely and appropriate follow‐up and there is a potential role for technology in the ear health pathway.
- Stakeholders recommended a range of strategies are needed, including strengthening workforce capacity and community education to raise awareness of the impacts of poor ear and hearing health.

## 1 Introduction

Aboriginal and Torres Strait Islander peoples flourished for 65 000 years in Australia prior to colonial invasion [1]. Colonisation has intentionally disrupted Aboriginal and Torres Strait Islander culture and wellbeing through assimilation policies, forcible removal of children, environmental dispossession and socioeconomic discrimination [2]. As a result, Aboriginal and Torres Strait Islander children in Australia experience some of the highest levels of ear disease globally [3]. Compared to non‐Indigenous children, Aboriginal and Torres Strait Islander children experience 8.5 times the burden of otitis media (41.9 vs. 4.9 DALYs per 100 000) [4]. Three times more Aboriginal and Torres Strait Islander children experience otitis media as a long‐term ear or hearing problem (2.6%) compared to non‐Indigenous children (0.9%) [5]. Ear and hearing health are critical to child wellbeing as they underpin communication, literacy, social skills and cognitive development [6, 7]. Ear disease and hearing impairment during childhood can have lifelong ramifications for language development, quality of life, educational attainment and interactions with the juvenile justice system [8]. Factors associated with increased otitis media occurrence—one of the most common childhood ear diseases—include overcrowding, social disadvantage, rural residency, passive smoking and poor access to health services [3, 9]. Fortunately, the World Health Organisation (WHO) estimates 60% of childhood hearing loss is preventable through public health action, including immunisation, newborn hearing screening, management of common ear diseases and childcare practices [10].

The impacts of colonisation on Aboriginal and Torres Strait Islander wellbeing are confounded by the inability of mainstream health services to meet the needs of Aboriginal and Torres Strait Islander communities. To overcome these service shortcomings, Aboriginal Community Controlled Health Services (ACCHS) were established in the 1970s with the aim to minimise health inequities experienced by Aboriginal and Torres Strait Islander peoples [11]. As part of this, ACCHS support Aboriginal and Torres Strait Islander self‐determination through accountability to community values and needs [12, 13]. Central to the success of ACCHS is the role of Aboriginal Health Workers and Practitioners (AHWP) who support various programmes across a range of health conditions, including ear and hearing health. Evidence has demonstrated that the involvement of AHWP in service delivery results in improved outcomes for Aboriginal and Torres Strait Islander peoples [14, 15].

In 2017, ‘An Aboriginal Ear Health Framework for South Australia’ was published by the South Australia Aboriginal Ear Health Reference Group (SAAEHRG), which included strategies to increase ear screening amongst Aboriginal and Torres Strait Islander children [16]. Central to these strategies was the importance of the AHWP workforce. This document recognised the importance of AHWP involvement in ear and hearing health screening and the need to ensure appropriate training and support is available for this workforce [17]. The implementation of ear and hearing screening programmes across South Australia occurs across several modalities and is funded in various, often inconsistent, ways. Despite national and state policy guidelines recommending involvement of AHWP in ear and hearing screening programmes [18], there has been no investigation into the supports already in place, nor those needed, at an organisational level to enable the implementation of these recommendations. The purpose of this study was to gather stakeholder perspectives on ear and hearing health screening across South Australia and the involvement, or potential involvement, of AHWP in these programmes with the intention for findings to inform strategies that optimise the role of AHWP in the delivery of ear and hearing screening programmes.

## 2 Methods

We conducted a qualitative study using semi‐structured interviews to gather the perspectives of key stakeholder informants regarding current ear and hearing health screening across South Australia. The study has been reported in accordance with the Consolidated Criteria for Reporting Health Research (COREQ) [19], and with consideration of the Centre of Research Excellence in Aboriginal Chronic Disease Knowledge Translation and Exchange (CREATE) tool (see Supporting Information) [20].

### 2.1 Leadership and Governance

To ensure respectful, reciprocal and culturally safe practices in the design and conduct of this project, the research was co‐led by an Aboriginal (LQ) and non‐Aboriginal (JHS) researcher in line with recommended practices [21]. During the design and establishment of the study, there was consultation with the SAAEHRG to get input on the design of the study and the interview guide. This research project received ethical approval from the Aboriginal Health Research Ethics Committee (HREC) (Reference: 04‐21‐921), the Southern Adelaide Local Health Network (SALHN) HREC (Reference: 2021/HRE00296) and the Flinders University HREC (Reference: 4099).

### 2.2 Eligibility Criteria

Inclusion criteria for this study included stakeholders who were over 18 years of age and had experience in senior policy, administrative or clinical roles within agencies involved in the delivery of ear health and hearing screening for Aboriginal and Torres Strait Islander children, either in South Australia or nationally. In recognition of the dominance of mainstream services in providing ear health and hearing screening, Aboriginal or Torres Strait Islander identity was not an inclusion criterion.

### 2.3 Recruitment

Purposive recruitment and snowball sampling [22] were employed in this study using existing networks to identify a diverse sample of stakeholders. Potential participants were initially contacted by the research team via email and invited to contact the researchers to receive more information if they were interested in participating. After a 2‐week period, potential participants who had not replied were sent a follow‐up email and asked to recommend a colleague who could participate in their place. All participants provided written informed consent to participate in the study, which was verbally reconfirmed at the commencement of the interview.

### 2.4 Interview Guide

A semi‐structured interview guide was developed to explore stakeholder perspectives of the barriers and facilitators influencing the implementation of the 2017 South Australian Ear Health Framework, as well as their views on the engagement of AHWP in ear health and hearing screening. A focus of the interviews was on exploring stakeholder views on how AHWP participation in ear health and hearing screening programmes can be better supported. Semi‐structured interviews were used to ask questions in an open‐ended and systematic way to allow interview participants to diverge from the main topic, as appropriate [23]. Open‐ended questions were followed up with both scripted and unscripted probes to enhance data captured within the semi‐structured interviews [24]. The semi‐structured interview guide was piloted prior to use and reviewed by the research leadership team (JHS, LQ, BFP), as well as the SAAEHRG membership. The guide is available in the Supporting Information.

### 2.5 Data Collection

All interviews were conducted via teleconference to accommodate the varying locations of the participants and the COVID‐19 restrictions in place at the time. Interviews were facilitated by the research assistant (BFP) using the semi‐structured interview guide. Field notes were not made after each interview; however, the interviewer discussed key concepts from each interview with members of the research team (JHS and LQ). Data collection continued until data saturation was reached, that is, no new concepts were raised by participants during interviews. Participants were offered small thank‐you gifts ($20 grocery gift vouchers) to recognise their time and contributions to the project. Interviews were audio recorded and transcribed verbatim by a contracted transcription service. Participants were offered the opportunity to review their transcript for comment and/or correction; however, all participants declined this offer, quoting a lack of time within their schedule as being prohibitive.

### 2.6 Researcher Positionality

Qualitative methodologies embrace the subjectivity of researchers interpreting findings and support researcher reflexivity [25]. Interviews with stakeholders, the subsequent thematic analysis was guided by an author (BFP) who is a non‐Indigenous researcher with a background in community‐based qualitative research with Indigenous communities in Canada and Australia. The thematic analysis was supervised by the project's primary investigator (JHS), who is a non‐Indigenous researcher with a background in mixed‐methods community‐based research. The research team was guided by the project's lead Aboriginal researcher (LQ). Two authors (PLM and NJS) are non‐Indigenous clinicians with experience providing paediatric ear and hearing healthcare to Aboriginal and Torres Strait Islander children, while one author (AM) is a non‐Indigenous researcher with a background in education.

### 2.7 Thematic Analysis

The analytic process was informed by Braun and Clarke's guidelines for reflexive thematic analysis [25, 26, 27]. Analysis of stakeholder interviews was completed by two researchers (BFP and JHS) and without a structured codebook, which enabled an iterative and organic process of thematic identification [25]. Interviews were inductively coded line by line within NVivo 12 software (QSR International Pty Ltd., Melbourne, Australia). The identified codes and themes were discussed amongst a core panel of researchers, which included both non‐Indigenous and Indigenous researchers (BFP, LQ, JHS). Data were re‐visited after initial analysis multiple times to permit the collation of similar codes and the development of themes [26]. Final themes were agreed upon following a roundtable consensus. These themes were mapped against a previously described ecological framework [28]. This process identified the themes as individual, local community, state or national level factors and supported the development of an understanding of how the themes intersect and influence each other.

## 3 Results

Between 9 May and 6 June 2022, we invited 32 stakeholders to participate in our study with 13 consenting to participate (41% consent rate). Reasons given for non‐participation were all related to time constraints and competing work commitments. All interviews with consenting participants were conducted via teleconference and ranged in duration from 14 to 50 min (mean: 24.2 ± 10.3 min). The mean age of the participants was 52.8 (± 10.9) years with three (23.1%) identifying as male. While participants worked across a range of sectors, the majority worked in the government sector (61.5%). Participants were primarily employed as managers (38.5%) or audiologists (30.8%). They reported working in healthcare for between four and 41 years (mean: 23.3 ± 12.1 years) and with an ear and hearing health focus for between one and 41 years (mean: 15.8 ± 10.7 years). Six (46.2%) of the participants had an audiology qualification and four (30.8%) identified as Aboriginal and/or Torres Strait Islander. One participant (8.3%) had lived experience of hearing impairment. The characteristics of the participants are presented in Table 1.

**TABLE 1:** Characteristics of the study participants (*N* = 13).

	*n*	%
Gender identity		
Male	3	23.1%
Female	10	76.9%
Other	0	0%
Current Employment Sector		
Community Controlled sector	1	7.7%
Government	8	61.5%
Industry/NGO	3	23.1%
University	1	7.7%
Current Primary Role		
Audiologist	4	30.8%
Manager	5	38.5%
Policy officer	2	15.4%
Lecturer	1	7.7%
Doctor	1	7.7%
Has an Audiology qualification		
Yes	6	46.2%
No	7	53.8%
Identifies as Aboriginal and Torres Strait Islander		
Yes	4	30.8%
No	9	69.2%
Lived experience of impaired hearing		
Yes	1	8.3%
No	12	91.7%

**FIGURE 1:**
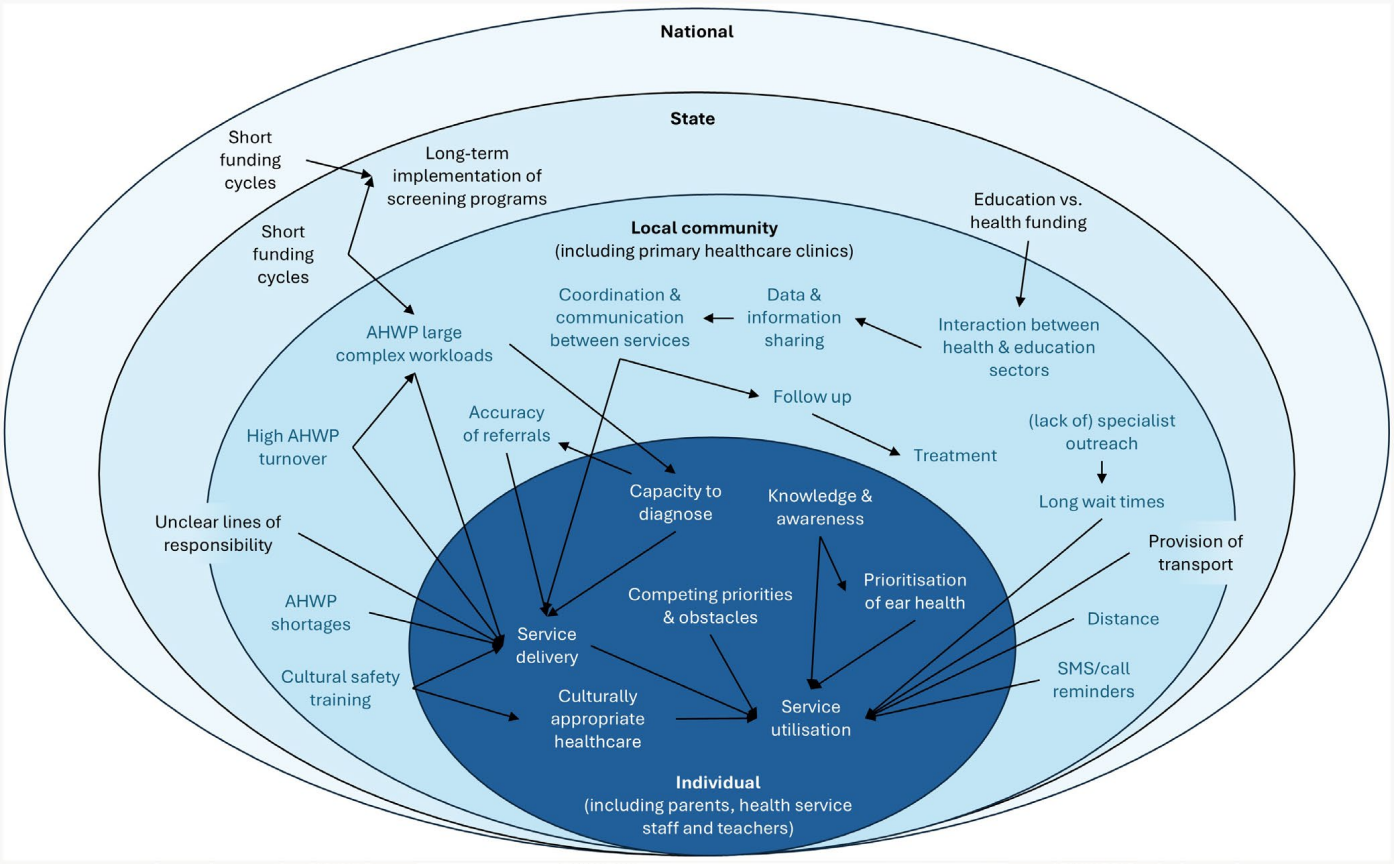
Themes and subthemes mapped to the Ecological Framework.

Stakeholder perspectives on ear and hearing health screening were categorised into five themes. These themes were: (i) a fragmented, short‐sighted and inconsistent system; (ii) confusion around scope of practice; (iii) workforce capacity; (iv) community awareness and knowledge and (v) service provision and utilisation. We provide key quotes to illustrate the themes and subthemes raised by stakeholders. Concepts from the themes and subthemes were mapped against the ecological framework (Figure 1). Most of the concepts were individual—at the parent/caregiver, staff or teacher—or local community level factors. The themes are explored in detail below with illustrative quotes provided in Table 2.

**TABLE 2:** Illustrative quotes for each subtheme generated from the interviews with stakeholders.

Theme	Subthemes	Quotes
Theme 1: A fragmented, short‐sighted and inconsistent system	Short funding cycles	*There were issues that an organisation would provide the screening for a period. And then, if funding stopped, they would stop screening* (Stakeholder 13). *There's all of the talk about how we need to screen them, but we don't actually have their commitment from above, we're asked to absorb it within our current program (Stakeholder 2)*.
	Coordination and communication between services	*There didn't seem to be any coordination of services, and that was the big thing. No one knew what the other team was doing. There's no communication across the networks, in the health networks here in Australia. They're all operating separately and doing their own thing. So I think, from what I remember, that the biggest thing for me was that there wasn't a clear, overarching thing that everyone was doing consistently. It's everyone was doing their own thing, their own project and yeah, no communication (Stakeholder 1)*.
	Inconsistencies in service provision	*Yeah, so I think there are some sites which are potentially over‐serviced by audiology, and some which are under … so I think there are some sites which are potentially over‐serviced by audiology, and some which are under. But because we don't have a way to connect the dots and connect the information, it's very, very hard to see what's happening in that child's ear health journey (Stakeholder 7)*.
Theme 2: Confusion around scope of practice	Synergy between health and education	*The interesting part with Education doing it – in some respects, this is a health issue. It's not, yes, it has an impact on education but, in some respects, we're spending education dollars on a health issue (Stakeholder 13)*. *… the Department of Education, they do school screenings as well, and there doesn't seem to be any coordination or interaction at all between the two groups [health and education], so they don't really know what the other is doing… (Stakeholder 1)*. *‘… if you ask what happened as a result of that screening visit, of those screening visits, often people can't answer what happened next for those kids. In my experience when I went up and talked to both schools and to health practitioners about what happened … they would have to be extremely proactive in following up to know what happened and if it made a difference’ (Stakeholder 4)*.
	Lines of responsibility	*Where does the scope of practice from an Aboriginal health practitioner who just does the screening, to then the audiologist who does the next steps, to the ENT who does the next step (Stakeholder 10)*.
Theme 3: Workforce capacity	Large complex AHWP workloads	*I know the intent is and people will say, every Aboriginal child at every occasion, look into their ears, but the ears are not the only thing, there are so many areas that require attention that every area is important. They will say at every opportunity check the child's skin. At every opportunity check the child's teeth. Ask about nutrition, ask about how they are going in life and how they're going in school? … there is a lot to check … And what happens is you hardly ever have an opportunity, because … unless they've got sore ears, … that's not the time that you're going to be checking their hearing (Stakeholder 8)*. *We've tried to bring the speed up, but we've trained all our Aboriginal health workers and practitioners in ear health and screening, but there's so much our practitioners and health workers need to do. Unfortunately ear health may not be as a priority as the presenting condition and therefore it will not get done (Stakeholder 6)*.
	Workforce shortages and high staff turnover	*100%. So, the biggest gap we find here in South Australia, is the supply of registered Aboriginal health practitioners (Stakeholder 6)*. *So, I think that there are breakdowns in—obviously in—and it will vary enormously from clinic to clinic and then of course, you know, there's churning in staff, a huge amount of turnover in the remote clinic staffing (Stakeholder 3)*.
	Culturally appropriate health care	*The most important thing is to deliver the service in a culturally appropriate and safe way. And I'm very conscious that quite a number of us, being educated in a non‐Aboriginal system and all that, and we're used to how we deliver services generally, just be mindful that that model is not—cannot just be replicated or duplicated or forced upon because there's no point having the best service if people don't feel comfortable coming (Stakeholder 5)*. *And the number of attempts that they had made to go and see private GPs to get something sorted out—the child was given antibiotics and that was it, there was no follow‐through, there was no recommendations to get the child's hearing tested—it was just fixing up that acute problem which was discharge at that time. I think it also reflected a lack of awareness and understanding within the broader medical workforce about ear disease in Aboriginal children. So, I think the people that he was talking about, the private GPs here, that he had taken the child to (? wasn't) behaving in line with what it would be for the general population, with the college of GPs guidelines were based on—I'm not saying that the GP did not do what they thought was right, it's just that their knowledge of what was right was where there was a problem. And they hadn't asked whether the child—they hadn't asked about the Aboriginal status, so they didn't know that this person was an Aboriginal kid (Stakeholder 8)*.
Theme 4: Community awareness and knowledge	Parents lack knowledge of impacts of ear disease	*Parents lack of understanding around how hearing impacts on the other domains of life, like education, speech development, behaviour, and all of those sort of key areas … the really big thing is quite often if kids aren't picked up early enough, by the time they see someone in the school system, they're already labelled as a disruptive kid or the kids that can't concentrate. Or there's a whole heap of other labels that have been put on that kid. And just that significant impact of, it can take years for kids to actually recover that missed time that they've had. And I think we underplay that a lot, even when we talk to parents about it. And I get like we don't want to overwhelm parents and caregivers, but we do need to be more proactive at the prevention stage by doing that conversation about, if your kid does end up with a significant ear health issue, this is what can happen, which is why we're doing this (Stakeholder 11)*.
	Schools lack awareness of ear disease	*Sometimes schools are just not aware, and have not attributed a lack of success or bad behaviour or disengagement on the fact that [students] may not be hearing (Stakeholder 13)*.
Theme 5: Service provision and utilisation	Non‐attendance	*We can provide the service and we can be there and do it all but, if we don't actually see the patients, it's not really working. And I find the non‐attendance rates are so high and I know it's always an issue, and it's been an issue forever and in every clinic with these populations, unfortunately. We provide transport, and we call and give them reminders the day before, and still people don't show up (Stakeholder 1)*.
	Long wait times and lack of follow‐up	*We screen thousands of kids a year through our service, and what we found was if we will find an ear abnormality that will need an audiology or an ENT referral, and these poor kids will be referred and they're lost in the system where there's high waits for audiology or high waits for ENT, and the only way we would know that these kids did get an intervention or not get an intervention is the next year we'll go back to that school and see that child has still the same tear in the ear drum, or something like that (Stakeholder 6)*.
	Competing priorities and multiple obstacles to overcome to attend appointments	*For that parent or guardian, that's not the only child that they've got things to deal with, there's a whole lot of else. So, [they're] prioritising because there is so much else going on in their lives. … But when you're talking about people who already have a lot that they're dealing with, that's one too many obstacles—if they have to take a child somewhere to get the screening done and then to get the results, and then see what happens out of that. And if you look at it in terms of your regular clinical work, and I'm only going to talk about Aboriginal clinical services, Aboriginal health services, I'm not even going to talk about mainstream because that's 10‐fold worse, because of the model (Stakeholder 8)*.
	Lack of specialist outreach	*there was no concomitant improvement in particularly good access to primary—or they have access to primary healthcare, but not primary healthcare that encompassed health and they certainly didn't have in those days, they didn't have access to specialist ear, nose and throat people, typically unless they actually went off the lands to somewhere like Port Augusta or something like that. Which is a massive undertaking (Stakeholder 3)*.

### 3.1 Theme 1: A Fragmented, Short‐Sighted and Inconsistent System

The current ear and hearing health screening landscape was seen as fragmented, short‐sighted and inconsistent. These issues were underpinned by clear shortcomings which could be explained by three subthemes: (i) short funding cycles; (ii) lack of coordination and communication between services and (iii) inconsistencies in service provision.

#### 3.1.1 Short Funding Cycles

Short funding cycles, a lack of coordination and communication between services and inconsistencies in service provision were identified by stakeholders as significant barriers to the delivery of screening services. Short funding cycles impede the long‐term implementation of screening programmes. Clinicians reported the expectation that screening would continue within their workload despite funding for screening programmes ending.

#### 3.1.2 Lack of Coordination and Communication Between Services

Poor coordination and communication between services were identified as a significant source of frustration for many stakeholders, with most expressing a desire for a more consistent approach. The quote in Table 2 illustrates the widespread nature of this fragmentation:

#### 3.1.3 Inconsistencies in Service Provision

Although not explicitly stated, participants alluded to poor communication and coordination between services, leading to inconsistent service provision. Participants stated they were ‘… keen not to duplicate each other's work’ but described a lack of data and information sharing that created an environment where ensuring consistent service delivery was difficult.

### 3.2 Theme 2: Confusion Around Scope of Practice

Additional barriers to the implementation of successful screening programmes, as identified by stakeholders, were the confusion about the scope of practice of different roles and organisations. These barriers could be clearly described by two themes: (i) a (lack of) synergy between health and education sectors and (ii) unclear lines of responsibility. These are discussed below.

#### 3.2.1 A (Lack of) Synergy Between Health and Education Sectors

Our cohort recognised the impact of ear health on education and the potential for the two sectors to work together synergistically, but also raised concerns that funding for education was being spent on a health issue. When reflecting on ear health screening being facilitated through the government's education department, one participant reflected: *‘it has an impact on education but, in some respects, we're spending education dollars on a health issue*’ (Stakeholder 13). There was also a perceived lack of coordination and interaction between health and education sectors. Data sharing between the health and education sectors was also described as lacking. There was a disconnect between the education sector‐led screening programme and the health sector follow‐up for children, with one participant stating: ‘*… if you ask what happened as a result of that screening visit, of those screening visits, often people can't answer what happened next for those kids*’ (Stakeholder 4).

#### 3.2.2 Unclear Lines of Responsibility

Participants also reflected on how, within a clinic or health service setting, there were potential issues with conflicting roles and responsibilities. The ability for AHWP to undertake screening as part of their core responsibilities may be influenced by administrative or managerial expectations on who is responsible for screening. Communication amongst staff, and when referral of a patient from one clinician to another should occur, may be unclear. One participant reflected on when responsibility shifts from one staffing role to another: ‘*Where does the scope of practice from an Aboriginal Health Practitioner who just does the screening, to then the audiologist who does the next steps, to the ENT who does the next step …*’ (Stakeholder 10).

### 3.3 Theme 3: Workforce Capacity

Clear workforce capacity issues were discussed by stakeholders. These were clearly delineated into the following three subthemes: (i) large complex AHWP workloads; (ii) workforce shortages and high staff turnover; and (iii) providing culturally appropriate health care. We discuss these themes in detail below.

#### 3.3.1 Large Complex AHWP Workloads

When stakeholders were asked how AHWP could be better supported to perform ear health screening, they explained that ear health screening may not be viewed as a priority due to AHWP having large complex workloads. Stakeholders explained AHWP are expected to perform a range of health checks, including those of ears, skin, teeth, nutrition and schooling, as part of their roles. Furthermore, unless a child presented with a known ear problem or a parent raised concerns, screening may not be performed. Stakeholders also discussed that despite widespread agreement in guidelines and amongst ear health practitioners that Aboriginal and Torres Strait Islander children should have their tympanic membrane (eardrum) viewed via otoscopy at every appointment, this does not occur, with the quote in Table 2 illustrating this perception. Furthermore, although health services provide training for their AHWP on how to undertake ear and hearing screening, due to the large workloads of AHWP, the reason for the child presenting to the clinic may be viewed as a higher priority, and, therefore, ears and hearing may not be screened. As stated in the following quote:

We've trained all our Aboriginal Health Workers and Practitioners in ear health and screening, but there's so much our practitioners and health workers need to do. Unfortunately, ear health may not be as [high] a priority as the presenting condition and therefore it will not get done (Stakeholder 6).

#### 3.3.2 Workforce Shortages and High Staff Turnover

A shortage of the AHWP workforce and high ACCHS staff turnover were identified by stakeholders as contributors to the lack of consistency in the provision of ear health screening services. In addition, more registered AHWP would be required to reduce the current high workload placed on AHWP, with one stakeholder stating the biggest gap was:

The supply of registered Aboriginal Health Practitioners … if we had enough of them, we could spread the load, but I think that's the biggest challenge … an already over‐worked workforce (Stakeholder 6).

Furthermore, stakeholders perceived there was high staff turnover in ACCHS, particularly in remote locations. As described by one participant (Stakeholder 3): ‘… *there's churning in staff, a huge amount of turnover in the remote clinic staffing*’.

#### 3.3.3 Culturally Appropriate Health Care

Stakeholders felt strongly about the importance of delivering culturally appropriate care for Aboriginal and Torres Strait Islander families and children. There was a strong consensus that mainstream models of healthcare and health worker training were insufficient and often inappropriate. Stakeholders reflected on how their own training and the health services where they worked were embedded in a Western, biomedical model of health and, as such, often did not align with culturally safe practices (see Table 2). Other stakeholders discussed culturally unsafe practices they had become aware of, which had occurred outside of the ACCHS sector. One stakeholder described a scenario where a child had been seen by a private general practitioner who was clearly unaware of the specialised guidelines for treating ear disease for Aboriginal and Torres Strait Islander children. Furthermore, the general practitioner had failed to provide culturally safe care as they had failed to ask about the Indigenous status of the child (see Table 2, quote from Stakeholder 8).

### 3.4 Theme 4: Community Awareness and Knowledge

Stakeholders identified two community subgroups who they perceived lack ear and hearing health awareness and knowledge. These were: (i) parents and (ii) school staff. We describe the perceived awareness and knowledge gaps below.

#### 3.4.1 Parents Lack Knowledge of Impacts of Ear Disease

Stakeholders expressed a perception parents (and caregivers) lack knowledge and awareness of ear disease and its impacts. While discussing this perceived gap, stakeholders stated there was a need to increase parental education and awareness on ear disease and its potential impacts. They stated that without increased levels of understanding, parents may not realise their child has an ear or hearing problem and expressed concern about the potential adverse impacts of this. One stakeholder gave an extensive description of this, which is provided in Table 2; however, an excerpt is provided:

… parents lack of understanding around how hearing impacts on the other domains of life, like education, speech development, behaviour, and all of those sort of key areas (Stakeholder 11).

#### 3.4.2 School Staff Lack Awareness of Ear Disease

Stakeholders also described their perception teachers and other school staff lacked awareness of ear disease. They expressed concern that teachers and school staff may not typically consider a child could be displaying poor behaviour or disengagement due to a hearing impairment. The implications of this were explored in the previous quote, that is, that children can be negatively labelled as disobedient, naughty, ‘disruptive’ or ‘can't concentrate’. See Table 2 for an illustrative quote.

### 3.5 Theme 5: Service Provision and Utilisation

The final theme in the stakeholder interviews encompassed four subthemes related to service provision and utilisation. These four subthemes were: (i) non‐attendance; (ii) long wait times and lack of follow‐up; (iii) competing priorities and multiple obstacles to overcome to attend appointments; and (iv) lack of specialist outreach services.

#### 3.5.1 Non‐Attendance

Stakeholders stated non‐attendance rates in both primary care and hospital settings across geographies (urban, rural and remote) were high. These high non‐attendance rates persisted despite providing transport and using phone call reminders prior to appointment times. In the following quote, a stakeholder shares their experience and states that the non‐attendance issue has been longstanding.

#### 3.5.2 Long Wait Times and Lack of Follow‐Up

Long waiting times and a lack of follow‐up care were described. Long waiting times were described by participants as the result of the intermittent ‘fly in‐out’ service provision by clinicians travelling to regional locations from metropolitan locations. The (in)frequency of this travel created long local waiting times, with children needing to wait to attend audiological and surgical services for diagnosis and treatment for their ear disease. Stakeholders were concerned about the potential for lack of, or loss to, follow‐up for children screened in a non‐health setting, such as within a school. This reflects and intersects with the previously described theme on the lack of synergy between health and education sectors. The quote in Table 2 illustrates the intersection of waiting times and follow‐up issues. Furthermore, the lack of follow‐up caused frustration for stakeholders, with one stakeholder asserting that screening should not take place unless there is capacity for timely and appropriate follow‐up care:

…if you can't action what comes out of the screening then you shouldn't be screening in the first place… (Stakeholder 8).

#### 3.5.3 Competing Priorities and Multiple Obstacles to Overcome to Attend Appointments

Stakeholders expressed that parents may not prioritise ear health screening due to competing priorities, as well as having multiple obstacles to overcome to attend clinic appointments. These two barriers to ear health screening were discussed in conjunction with attendance rates, and it is inferred that these were key reasons for clinic non‐attendance (see Table 2 for illustrative quotes).

#### 3.5.4 Lack of Specialist Outreach

Stakeholders stated access to specialist services was impeded by the need for parents and caregivers to travel long distances to access these services for their children. This was particularly pertinent for those families who resided in remote locations and had to travel many hours for ear and hearing healthcare due to a lack of local, in‐community specialist outreach services. The following quote provides an illustration of this:

… they didn't have access to specialist ear, nose and throat people, typically unless they actually went off the lands to somewhere like Port Augusta or something like that. Which is a massive undertaking (Stakeholder 3).

### 3.6 Recommendations From Stakeholders

Priority areas and strategies for improvement suggested by stakeholders are summarised in Table 3. Three overarching recommendations were suggested. Firstly, stakeholders identified the need to strengthen the capacity of the ear and hearing health workforce. Within this were four components: (i) the need for training on how to interpret ear screening test results to improve the accuracy of referrals, (ii) the need for formal opportunities for AHWP to undertake ear health screening training, (iii) the need for informal opportunities for AHWP ear health screening training and (iv) an increased number of AHWP staff. Secondly, stakeholders identified the need to strengthen existing systems and ways of doing things by incorporating: (i) a family‐friendly approach, (ii) student placements, (iii) dedicated ear health champions, (iv) technology and (v) fast‐track pathways and rapid referral options. Finally, stakeholders recommended increased community education to raise awareness of the implications of poor ear and hearing health, particularly amongst the (i) general population and (ii) educators.

**TABLE 3:** Priority areas identified by stakeholders to be addressed to improve ear and hearing healthcare for aboriginal and torres strait islander children.

Priority area	Strategies for improvement	Quotes
Strengthening workforce capacity	Increase training to improve accuracy of referrals	*I think more training, I think, is a big thing. For us, we're finding that some of the results are a bit inconsistent or the screeners are saying that they haven't passed when they have. So I think there are a few consistencies and double‐ups where we might not necessarily need to see someone, they get referred anyway. (Stakeholder 1)*
	Formal opportunities for AHWP ear health screening training	*It's more than that. Yes and no, but it's almost like, ‘Okay, I've gone and done my qualifications and I've got this generic [certificate]’. It'd be like, ‘I've gone and done my nursing and I've got this generic qual, I'm now a registered professional and working in that space’. I actually really enjoy—or I really see a lot of ears stuff going on here. How do I get my skill set up? How do I improve? I know Ear Health here in Australia has, but they're not skill sets under my quals that I can then add to my profession and be recognised in that space. (Stakeholder 10)*
	Informal opportunities for AHWP ear health screening training	*And so it was peer knowledge, I guess you could probably call it. I don't know if there is a formal training; there probably is, I imagine, but for us, I think it's always been peer knowledge, what to look for. (Stakeholder 9)*
	More AHWP staff	*I think it's so important that having the Aboriginal health care workers so involved is key, because I see how [AHWP] is at the clinic. The patients love him, and they really connect with him and they respect him, they want him in an appointment and things like that. And it's so valuable, having people like that, who the patients connect with, because I think they'll really trust someone, and then listen to them, hopefully, if they give them information. (Stakeholder 1)*
Strengthening systems and ways of doing things	Family friendly approach	*So the fact that it was Independent from the family perspective and not necessarily adhering to what the criteria was, or I kind of was open to relaxing the criteria for people being screened including increasing the age group in having all the children in the family, because if you're doing it the Aboriginal way, you've got to think about doing the whole family‐friendly way. (Stakeholder 8)*
	Student placements	*Yeah, so what we did because we're very aware of the challenges of funding but also the challenges for universities and finding really, meeting placement. And so I came up with the notion that if I could negotiate for there to be placements, and I couldn't negotiate for some support from my agency, for me to facilitate a visit, that we might be able to set something up that would be sustainable. (Stakeholder 12)*
	Dedicated ear health champions to ensure timely follow‐up	*But I guess you have to have someone who will do that chasing and have someone employed whose responsibility it is and Johnathon, is pretty good at that and he'll have long conversations with key stakeholders about prompting ideas and helping them to articulate things and helping them to report on things. So, yeah, I guess it's who can do that would be a key thing. (Stakeholder 4)*
	Technology	*And so you know, if they're doing a video otoscopy and sending those results and the tympanometry results electronically, you know, back to a specialist who's giving advice to the clinic in terms of treatment and monitoring everything. I think that that—those are the way the role should probably proceed, where a lot of the decision‐making is taken and then not trying to be in any way belittling [00:34:24] health worker. (Stakeholder 3)*
	Fast track pathways and rapid referral	*I think we have a health system that is not functioning particularly well anywhere, but if you are able to pick out the kids who are at risk of delay, then maybe that offers a way of getting them fast tracked to care. So I know that some ENTs are starting to use that approach as well where they're going, ‘Okay, this kid is at risk of delay. We need to get them in faster’. Then that potentially offers a way of getting some kids faster tracked. (Stakeholder 4)*
Community education	Public education	*I think public education to emphasise the importance of good ear health and hearing health and the impact on the future and impact on the potential of the children. (Stakeholder 5)*
	Teacher education	*… But also, to increase awareness for teachers and early educators in that space around hearing and language development, but also to provide some upskilling opportunities. (Stakeholder 11)*

## 4 Discussion

Largely due to the ongoing impacts of colonisation and biomedical models of care, the ear and hearing health of Aboriginal and Torres Strait Islander children is amongst the worst in the world. National and state policy guidelines recommend involving AHWP in ear and hearing health as a mechanism to improve the identification and treatment of children with ear disease. Herein, we present the first study to explore stakeholder perspectives on ear and hearing health screening across South Australia, including how AHWP can be better supported to deliver ear and hearing screening programmes and strategies to improve service delivery. Stakeholders identified five key themes which influenced the success of ear and hearing screening in South Australia, as well as factors relevant to the involvement of AHWP in these processes. The themes identified in the stakeholder interviews highlight a complex interplay between all levels of the ecological model. These factors can be targeted to improve access to timely and appropriate diagnosis and treatment of otitis media to prevent negative sequelae.

The first theme was that the current service provision was fragmented, short‐sighted and inconsistent and stated this contributes to inconsistent service provision. Our findings reflect previously described areas of the Australian universal healthcare system where there are gaps for some populations. A recent systematic review found that fragmentation of health care delivery and inequity in access to care were key issues across the entire Australian health system [29]. More specifically, for Aboriginal and Torres Strait Islander people living in regional and remote Australia, child health services could be improved by enhanced communication and coordination between providers [28, 30].

Secondly, confusion exists on clinicians' scope of practice, particularly for AHWP, and a disconnect exists between the health and education sectors on roles and responsibilities. In South Australia, there has been a longstanding collaborative school‐based ear health screening programme delivered in partnership by the education and health sectors. The importance of strengthening this ongoing partnership was identified as a priority in the 2012 SAAEHRG Framework [16]. The Framework also included recommendations for the adoption of a standardised, consistent approach to care [16]; echoing a recommendation made in the *European Consensus Statement on Hearing Screening of Pre‐school and School‐age Children* [31]. However, over a decade later there continues to be an inconsistency in how ear health screening is locally implemented, which reflects the ongoing global lack of standardised protocols and guidelines on how ear health screening is best implemented in school settings [32]. This likely reflects the ongoing debate about the benefit of population‐wide screening of asymptomatic children [33], despite evidence suggesting school‐based hearing screening programmes are cost‐effective [34]. As described by our cohort, school‐based screening programmes also have insufficient data capture processes for monitoring the long‐term outcomes of the programme, as well as insufficient documentation of referral procedures to ensure children requiring follow‐up are connected to appropriate diagnostic and treatment services. While stakeholders identified these issues, none suggested strategies to overcome these barriers, which points to the complexity of the problem.

The third theme encompassed concerns about workforce capacity in both ACCHS and mainstream health services. Both mainstream services and ACCHS were identified as lacking workforce capacity, including high staff turnover and staff shortages. High rates of staff turnover and use of short‐term staff are known to decrease the effectiveness of healthcare and, ultimately, are less cost‐effective [35]. Reasons identified in the literature for difficulties retaining Indigenous health professionals include unsupportive work environments [35, 36], heavy workloads [36], unclear roles and responsibilities [36], lack of ongoing professional development [35], and low salary or a perception of salary disparity [36]. Amongst our cohort and reflecting the Framework recommendations [16], our cohort agreed AHWP involvement in ear health screening was beneficial, but there was a need to strengthen workforce capacity, including upskilling AHWP and recruiting more AHWP staff and staff shortages and associated large workloads needed to be addressed.

Our cohort of stakeholders identified the need for improved cultural safety across both mainstream services and ACCHS. Caregivers of Aboriginal children have emphasised the importance of culturally secure care [37, 38] with fear of racism, disrespect, judgement and negative government interventions identified as barriers to accessing some mainstream health services [28]. Training in cultural safety for non‐Indigenous healthcare staff must be mandated as it has clear implications for Aboriginal and Torres Strait Islander peoples' access and utilisation of health services. Going beyond cultural awareness, cultural safety ‘*enables safe [healthcare] service [as] defined by those who receive the service*’ [39]. Furthermore, the inclusion of AHWP in healthcare delivery facilitates trust between non‐Indigenous healthcare professionals and Aboriginal and Torres Strait Islander patients, thus enabling better access to care [28].

The fourth theme was focussed on the lack of awareness and knowledge around the signs and impacts of ear disease. Parents and school staff were thought to lack knowledge and awareness of ear disease and its associated impacts, suggesting the health literacy of educators and families warrants attention. This aligns with prior research in which parents and caregivers report they never received information about ear health and were unaware of the signs of otitis media, nor of its implications if left untreated [37]. Furthermore, these parents were more likely to attribute a child not listening to behavioural problems rather than a hearing impairment [37]. The long‐term implications of extended periods of hearing loss, particularly during key developmental periods in early childhood, are profound and can have detrimental impacts on auditory processing, listening skills, language and speech development, school readiness, social competence, psychosocial wellbeing and sleep [40]. Therefore, it is unsurprising that stakeholders advocated for the need for community education, particularly for teachers and parents, to raise awareness of the implications of poor ear and hearing health.

The final theme discussed by stakeholders concerned limitations in service provision and utilisation. High rates of non‐attendance were reported by our study cohort, with non‐attendance persisting despite services providing transport and phone call reminders. Stakeholders stated non‐attendance was contributed to by parents' competing priorities, lack of knowledge of the signs of otitis media and prior experiences of culturally unsafe care. Elsewhere, similar ‘life issues’ [28], out‐of‐pocket costs [41], and long wait times to see specialists [42] have been shown to impact on appointment attendance. Non‐attendance to post‐screening appointments has implications for the continuity of care for the child. The benefits of screening are lost if there are no appropriate and clear pathway mechanisms for follow‐up [16]. Dedicated ear health champions were recommended with evidence from other locations suggesting they are a successful strategy to facilitate timely and appropriate follow‐up [43, 44]. Moreover, caregivers have expressed shame and fear about seeking healthcare, concerned they may be reported to the government or have their child removed from their care if presented too often with a sick child [42]. However, evidence suggests carers would feel empowered to seek help if they were equipped with appropriate ear and hearing health knowledge [37]. These suggestions to implement a family‐friendly approach align with the SAAEHRG Framework [16] recommendation to acknowledge the multiple competing demands on families and communities, and the importance of family engagement and choice. Finally, stakeholders also suggested the potential role of technology in the ear and hearing healthcare, highlighting the potential for telehealth to facilitate increased appointment attendance. Telehealth for hearing and ENT appointments has been shown to be cost‐effective and reduce the waiting time for appointments [45]. Evidence also shows that the use of real‐time video‐otoscopy during telemedicine consultations is possible and provides better clinical care during the telehealth appointment [46]. As such, the role of telehealth in the delivery of ear and hearing healthcare will no doubt increase in future [47].

### 4.1 Strengths and Limitations

This research evolved from SAAEHRG‐driven queries about implementation barriers for the Framework's screening strategies and about the ways to increase AHWP involvement in ear health screening. Furthermore, the project received in‐principle support and interview guide input from members of the SAAEHRG. As a result, a key strength of this research is that it has direct policy and practice implications. Another strength was that the research prioritised six core values of research involving Aboriginal and Torres Strait Islander Peoples, including reciprocity, respect and responsibility [48, 49], with leadership from an Aboriginal researcher (LQ) and members of the SAAEHRG. However, as with any research, there are potential limitations to be acknowledged. Firstly, due to the impacts of COVID‐19, interviews were unable to be conducted in person and were instead conducted online using teleconference software. However, this opened an opportunity to be more flexible in the scheduling of interviews and the inclusion of stakeholders from interstate. Furthermore, while the stakeholder cohort may be considered small, we aimed to recruit a representative sample and to continue recruitment until data saturation was achieved. Data saturation was achieved quickly, and the final cohort is a representative group of stakeholders from a variety of government, not‐for‐profit and ACCHS organisations located within South Australia, as well as elsewhere nationally. Finally, while our findings have been generated in relation to the South Australian experience, the findings are likely to be representative of experiences both across Australia and in other Indigenous settings globally. Also, we note herein that there is an absence of AHWP voices; however, this data was collected and will be reported elsewhere.

### 4.2 Implications for Practice

This study generated evidence on the factors which support the implementation of ear and hearing screening programmes in an Australian population. Our findings can be used by policymakers to influence policy and practice change, including decision‐making about budget allocations. Our results highlight a complex interplay between all levels of the ecological model which requires targeting if access to timely and appropriate diagnosis and treatment of otitis media is to be improved for Aboriginal and Torres Strait Islander communities. At the individual level, this includes training AHWP to confidently and competently conduct ear health screening, training of non‐Indigenous healthcare staff to deliver culturally safe healthcare and increasing knowledge and awareness of ear health amongst the community and educators. At the local community level, an increased number of AHWP need to be employed as a strategy to reduce large and complex workloads, an increase in communication and data sharing within and between health and education sectors and addressing the lack of specialist outreach in rural and remote locations. At the state and national levels, longer‐term funding cycles would ensure continuity of services and stabilisation of the workforce. Using these findings as the impetus for policy and practice change will benefit communities by increasing early detection of ear disease and hearing impairment, leading to a reduction in long‐term burden on health and the associated hearing‐related developmental outcomes.

## 5 Conclusion

Our study reveals a complex interplay of factors impacting on ear health and hearing service access and utilisation for Aboriginal and Torres Strait Islander children at all levels of the ecological model. Stakeholders suggested several strategies to enable better access and utilisation of ear and hearing health services for Aboriginal and Torres Strait Islander children, which can be incorporated into current service delivery models to enhance service access and utilisation. Given the disproportionately high rates of otitis media amongst Aboriginal and Torres Strait Islander children, there is an urgent need to implement evidence‐based, community‐driven strategies to reduce the burden of ear disease, which will contribute to improved education and overall health and wellbeing outcomes for this population. Future research should focus on implementing and evaluating the effectiveness of these strategies over time.

## Author Contributions

**Jacqueline H. Stephens:** conceptualisation, funding acquisition, data curation, data analysis, project administration, methodology, supervision, writing – draft preparation, review and editing. **Brianna F. Poirier:** data curation, project administration, data analysis, methodology, writing – review and editing. **Patricia L. Macfarlane:** conceptualisation, funding acquisition, writing – review and editing. **Nicola J. Spurrier:** conceptualisation, funding acquisition, writing – review and editing. **Leanne Quirino:** conceptualisation, funding acquisition, data analysis, writing – review and editing.

## Funding

This research was supported by grants from the Flinders Foundation and Channel 7 Children's Research Foundation.

## Ethics Statement

This research project received ethical approval from the Aboriginal Health Research Ethics Committee (Reference: 04‐21‐921), the Southern Adelaide Local Health Network (SALHN) Human Research Ethics Committee (HREC) (Reference: 2021/HRE00296) and the Flinders University HREC (Reference: 4099).

## Consent

All participants provided written informed consent and reaffirmed their consent to participate at the commencement of each interview.

## Conflicts of Interest

The authors declare no conflicts of interest.

## Supporting information


**FILE 1** Consolidated criteria for reporting qualitative research (COREQ) checklist.
**FILE 2:** Aboriginal and Torres Strait Islander Quality Appraisal Tool.
**FILE 3:** Semi‐structured Interview Guide.

## Data Availability

The data that support the findings of this study are unavailable due to restrictions applied by the research ethics committees, and so are not publicly available.
