# Supplementary material for: Stakeholder Perspectives on Ear and Hearing Health Service Provision for Aboriginal and Torres Strait Islander Children
Source: Aust J Rural Health. 2026 Mar 25;34(2):e70167. doi: 10.1111/ajr.70167 (PMC13014056; doi:10.1111/ajr.70167)
Supplement: Supplementary file 1 — FILE 1 Consolidated criteria for reporting qualitative research (COREQ) checklist. FILE 2: Aboriginal and Torres Strait Islander Quality Appraisal Tool. FILE 3: Semi‐structured Interview Guide. [file AJR-34-0-s001.pdf]

SUPPLEMENTARY MATERIAL

Table of Contents

SUPPLEMENTARY FILE 1: Consolidated criteria for reporting qualitative research (COREQ) checklist

SUPPLEMENTARY FILE 2: Aboriginal and Torres Strait Islander Quality Appraisal Tool

SUPPLEMENTARY FILE 3: Semi-structured Interview Guide

COREQ (COnsolidated criteria for REporting Qualitative research) Checklist

A checklist of items that should be included in reports of qualitative research. You must report the page number in your manuscript where you consider each of the items listed in this checklist. If you have not included this information, either revise your manuscript accordingly before submitting or note N/A.

Topic	Item No.	Guide Questions/Description	Reported on Page No.
**Domain 1: Research team and reflexivity**			
Personal characteristics			
Interviewer/facilitator	1	Which author/s conducted the interview or focus group?	5
Credentials	2	What were the researcher's credentials? E.g. PhD, MD	5
Occupation	3	What was their occupation at the time of the study?	5
Gender	4	Was the researcher male or female?	5-6
Experience and training	5	What experience or training did the researcher have?	5-6
Relationship with participants			
Relationship established	6	Was a relationship established prior to study commencement?	5-6
Participant knowledge of the interviewer	7	What did the participants know about the researcher? e.g. personal goals, reasons for doing the research	5
Interviewer characteristics	8	What characteristics were reported about the inter viewer/facilitator? e.g. Bias, assumptions, reasons and interests in the research topic	5-6
**Domain 2: Study design**			
Theoretical framework			
Methodological orientation and Theory	9	What methodological orientation was stated to underpin the study? e.g. grounded theory, discourse analysis, ethnography, phenomenology, content analysis	6
Participant selection			
Sampling	10	How were participants selected? e.g. purposive, convenience, consecutive, snowball	4-5
Method of approach	11	How were participants approached? e.g. face-to-face, telephone, mail, email	4-5
Sample size	12	How many participants were in the study?	6
Non-participation	13	How many people refused to participate or dropped out? Reasons?	6
Setting			
Setting of data collection	14	Where was the data collected? e.g. home, clinic, workplace	5
Presence of non-participants	15	Was anyone else present besides the participants and researchers?	n.a.
Description of sample	16	What are the important characteristics of the sample? e.g. demographic data, date	6
Data collection			
Interview guide	17	Were questions, prompts, guides provided by the authors? Was it pilot tested?	5
Repeat interviews	18	Were repeat inter views carried out? If yes, how many?	n.a.
Audio/visual recording	19	Did the research use audio or visual recording to collect the data?	5
Field notes	20	Were field notes made during and/or after the inter view or focus group?	5
Duration	21	What was the duration of the inter views or focus group?	6
Data saturation	22	Was data saturation discussed?	5-6
Transcripts returned	23	Were transcripts returned to participants for comment and/or	5

Topic	Item No.	Guide Questions/Description	Reported on Page No.
		correction?	
**Domain 3: analysis and findings**			
Data analysis			
Number of data coders	24	How many data coders coded the data?	6
Description of the coding tree	25	Did authors provide a description of the coding tree?	n.a.
Derivation of themes	26	Were themes identified in advance or derived from the data?	6
Software	27	What software, if applicable, was used to manage the data?	6
Participant checking	28	Did participants provide feedback on the findings?	n.a.
Reporting			
Quotations presented	29	Were participant quotations presented to illustrate the themes/findings? Was each quotation identified? e.g. participant number	7-12, Tables 2 [illegible]
Data and findings consistent	30	Was there consistency between the data presented and the findings?	7-12
Clarity of major themes	31	Were major themes clearly presented in the findings?	7-12
Clarity of minor themes	32	Is there a description of diverse cases or discussion of minor themes?	7-12

Developed from: Tong A, Sainsbury P, Craig J. Consolidated criteria for reporting qualitative research (COREQ): a 32-item checklist for interviews and focus groups. *International Journal for Quality in Health Care*. 2007. Volume 19, Number 6: pp. 349 – 357

Once you have completed this checklist, please save a copy and upload it as part of your submission. DO NOT include this checklist as part of the main manuscript document. It must be uploaded as a separate file.

ABORIGINAL AND TORRES STRAIT ISLANDER QUALITY APPRAISAL TOOL

ABORIGINAL AND TORRES STRAIT ISLANDER QUALITY APPRAISAL TOOL

SELF ASSESSMENT

CREATE

Stakeholder Perspectives on Ear and Hearing Health Service Provision for Aboriginal and Torres Strait Islander Children.

Question	Yes	Partially	No	Unclear
1. Did the research respond to a need or priority determined by the community?	✓			
2. Was community consultation and engagement appropriately inclusive?	✓			
3. Did the research have Aboriginal and Torres Strait Islander research leadership?	✓			
4. Did the research have Aboriginal and Torres Strait Islander governance?	✓			
5. Were local community protocols respected and followed?	✓			
6. Did the researchers negotiate agreements in regards to rights of access to Aboriginal and Torres Strait Islander peoples' existing intellectual and cultural property?	✓			
7. Did the researchers negotiate agreements to protect Aboriginal and Torres Strait Islander peoples' ownership of intellectual and cultural property created through the research?	✓			
8. Did Aboriginal and Torres Strait Islander peoples and communities have control over the collection and management of research materials?		✓		
9. Was the research guided by an Indigenous research paradigm?			✓	
10. Does the research take a strengths-based approach, acknowledging and moving beyond practices that have harmed Aboriginal and Torres Strait peoples in the past?	✓			
11. Did the researchers plan to and translate the findings into sustainable changes in policy and/or practice?	✓			
12. Did the research benefit the participants and Aboriginal and Torres Strait Islander communities?	✓			
13. Did the research demonstrate capacity strengthening for Aboriginal and Torres Strait Islander individuals?	✓			
14. Did everyone involved in the research have opportunities to learn from each other?	✓			

SEMI-STRUCTURED INTERVIEW GUIDE

Key Stakeholders

Script

Hi, I'm <insert name>. Thanks for your time today. I am conducting this study as part of my work to develop strategies to optimise screening for ear disease and hearing impairment among Aboriginal and Torres Strait Islander children. I am interested in your views and ideas on what works well for ear screening and what could work better. This information will be used to inform improvements to the South Australian Ear Health Framework, as well as other community-driven strategies.

A little bit of time has passed since you spoke with <insert name> and signed the consent form. Do you have any questions about participating today?

I will start the study in a moment, but I want you to know we can stop the session at any time, so if you feel uncomfortable or are not happy with any of the questions, just let me know.

This interview will take between half an hour to one hour of your time, and I will be recording the interview so I can review our discussion later. I am going to start the recording now.

[Start voice recorder]

1) *Can I please re-confirm you consent to participate in this interview today?*
2) *Can I please re-confirm you consent for the interview to be audio-recorded for research purposes?*

3) *The first thing I would like to ask is for you to tell me a bit about your role at this organisation?*

4) *What's the first thing you think of when I say 'ear health'?*

5) *Does ear health impact on the kids in your community? How does it impact on them?*

6) *What impact do you think ear disease has on childhood development?*
Prompts: education, social and emotional wellbeing, employment

7) *What impact do you think this has later in life?*
Prompts: education, social and emotional wellbeing, employment

8) *I'm really interested in your experiences in doing ear health screening. Can you tell me about how ear health and screening gets done at the clinic where you work?*
Prompts: Do you do screening? Is there anything you would change in how it works? Can you tell me about the equipment being used? If you don't do screening, would you like to?

9) *Some Aboriginal Health Workers have done ear screening training. Have you or any of your staff ever done any training, and can you tell me about the training you/they received?*
Prompts: What did you/they learn? Was the training useful? How could it be improved?

10) *Sometimes it can take a long time for kids to get hearing checks or see an ear specialist. How do you think ear screening and access to ear health services can be improved?*
Prompts: navigators, health information, support for families

11) *In 2017 the South Australian Aboriginal Ear Health Reference Group published the South Australian Ear Health Framework. Are you familiar with this document? Do you think this document appropriately reflects the way ear health screening is done in South Australia?*

12) *In the Framework, Aboriginal Health Workers are identified as being key frontline workers well placed to perform ear health screening. Do you have any thoughts or comments on how Aboriginal Health Workers can be better supported to participate in the screening pathway?*

13) *Do you have any other thoughts on how the Framework can be improved? Are there any gaps? Any information missing or information that can be removed?*

Thank you for your time today. Do you have any other comments you would like to share?

I will end the recording now.

[Turn off recorder]
